# Acoustic emission applied to stochastic modeling of microdamage in compact bone

**DOI:** 10.1007/s10237-024-01838-2

**Published:** 2024-03-29

**Authors:** D. Sánchez-Molina, S. García-Vilana

**Affiliations:** 1 UPC-EEBE, GiES, Eduard Maristany, 14, 08036 Barcelona, Spain; 2 UPC-EEBE, GiES, Av. Víctor Balaguer, 11, 08800 Barcelona, Spain

**Keywords:** Acoustic emission, Biomechanics, Thorax injury, Human rib, Stochastic failure models, Cox process, Explosive stochastic process

## Abstract

Exploring the stochastic intricacies of bone microstructure is a promising way to make progress on the practical issue of bone fracture. This study investigates the fracture of human complete ribs subjected to bending and using acoustic emission (AE) for microfailure detection. As the strain increases, the number of AE signals per unit of time rises until, beyond a certain threshold, an avalanche of signals occurs, indicating the aggregation of numerous microfailures into a macroscopic fracture. Since microfailures appear randomly throughout the bending test, and given the lack of a deterministic law and the random nature of microfailures during the bending test, we opted to develop a stochastic model to account for their occurrence within the irregular and random microstructure of the cortical bone. Notable discoveries encompass the significant correlation between adjusted parameters of the stochastic model and the total number of microfailures with anthropometric variables such as age and body mass index (BMI). The progression of microfailures with strain is significantly more pronounced with age and BMI, as measured by the rate of bone deterioration. In addition, the rate of microfailures is significantly impacted by BMI alone. It is also observed that the average energy of the identified AE events adheres to a precisely defined Pareto distribution for every specimen, with the principal exponent exhibiting a significant correlation with anthropometric variables. From a mathematical standpoint, the model can be described as a double Cox stochastic and explosive (coxplosive process) model. This further provides insight into the reason why the ribs of older individuals are considerably less resilient than those of younger individuals, breaking under a considerably lower maximum strain ($\varepsilon _{\max }$).

## Introduction

The prediction of mechanical failures in bone tissue under stress is a problem of practical interest. Microdefects and random details of bone microstructure have a decisive effect on the occurrence of microcracks and the propagation of macroscopic cracks, according to a substantial body of recent research (O’Brien et al. 2005; Hoc et al. 2006; Wang et al. 2017; Jerban et al. 2020; Velázquez-Ameijide et al. 2020). For this reason, the ultimate breaking stress of different specimens of human compact bone presents some random variation, and at this point, *stochastic models* play an important role in elucidating many aspects of bone fracture (Najafi et al. 2007; Schechner et al. 2010; Arregui-Dalmases et al. 2015), as happens in other areas of the natural and engineering sciences (Pinsky and Karlin 2010; Jia and Gardoni 2018). The inherent variability in biological and medical procedures has been analyzed using models of this type in some past studies (Melzer et al. 2017; Sánchez-Molina et al. 2020).

The measures used to assess microdamage in this study are based on detection of Acoustic Emission (AE) events, which can be used to quantify the number of microfailures and could potentially be applied to *in vivo* monitoring of bone under stress (Shrivastava and Prakash 2009; Agcaoglu and Akkus 2013). The AE technique, which is non-interfering, has been utilized for a variety of purposes in the biomedical field in the past. In orthopedics, AE has been used for implant design, failure prediction or even orthopedic diagnosis (Kapur 2016; Khokhlova et al. 2020), and it has also been used real-time monitoring of various materials (Carpinteri et al. 2013; Agelis et al. 2016), including biological materials (Watanabe et al. 2001; Ampadi Ramachandran et al. 2022). Some biomedical studies analyzed the loading process of specific bones such as the femur or the tibia (Aggelis et al. 2015; Strantza et al. 2015), but most of these researches focused on the monitoring of damage under increasing load, and also on the influence of age and the distribution of energies and amplitudes of the recorded events (Sánchez-Molina et al. 2015; Baró et al. 2016). In most cases, no specific models were proposed to describe the occurrence of AE events as stress increases (García Vilana et al. 2016a, b). The model presented in this study is an new attempt to fill this gap.

In the framework of biomechanical applications, the AE technique has been used to analyze the fracture of bone and other biological materials; however, there are few quantitative models which use AE data for the prediction of fracture (Johansen and Sornette 2000; Davidsen et al. 2021; García-Vilana et al. 2022; Adrover-Monserrat et al. 2023). The novel part of this work lies in the stochastic model presented in Sect. 2.2, whose application to experimental data is discussed in Sect. 3.

## Materials and methods

In this section, the proposed stochastic failure model is presented. This model has been applied to data obtained from complete human rib bending tests until failure, which were monitored with the acoustic emission technique.

The structure of this section is as follows. First, the origin and handling of the specimens is described in 2.1, followed by the main part, a description of the stochastic model in 2.2. After this, the details of the procedure and data processing of the three-point bending tests in 2.2.1 and acoustic emission technique are presented 2.4. Finally, some comments on statistical procedures are presented in 2.5.

### Material

The material used to test the usefulness of the stochastic model of failure consisted of fresh human rib specimens harvested from forensic autopsies. All the specimens were initially removed for complementary medical-legal investigation. This study was approved by the Research and Ethics Committee of the IMLCFC. Fifteen healthy complete fourth ribs were obtained from autopsies of *post mortem* human subjects (PMHS). The average age was $50.9\pm 10.9$ years (= average ± StdDev, from 26 to 62 years) and Body Mass Index (BMI) was $32.6\pm 6.2$ kg/m^2^ (from 24.2 to 42.7 kg/m^2^). Prior to the experimental tests, the soft tissue and cartilage were removed.

l The data used in this study are available in the Institutional Repository CORA-RDR [https://dataverse.csuc.cat/], search under the name “Acoustic emission data of fractures in human ribs”.

### Stochastic model for microfailures

This section presents the stochastic model in an informal way, in order to not hide simple ideas behind notations and terminology. Some aspects of the model are derived from prior models employed to simulate the progressive deterioration of biological tissues (Sánchez-Molina et al. 2015), or to examine deterioration issues and machine maintenance (Yeh 1988).

The model describes probabilistically the progression of internal damage, by accumulation of microfailures and microcracking within the material. This progression is modeled by a *stochastic process* formed by a collection of random variables $\{N_{\varepsilon }\}_{\varepsilon \ge 0}$, which can be thought of as a one-dimensional stochastic process parametrized by $\varepsilon$ (see the “Appendix”, Sect. 5, for the mathematical details and definitions of stochastic processes). In this case, $\varepsilon$ corresponds to the tensile strain achieved, and $N_{\varepsilon }$ represents the number of microfailures detected when reaching the strain $\varepsilon$ (this number is correlated with the number of AE events detected). Microfailures occur at random over time in accordance with a non-homogeneous renewal Markov stochastic process. The value of the strain when the *k*-th microfailure appears is denoted as $\varepsilon _k$ (i.e.,, $N_{\varepsilon _k} = k$) and the strain increment between the *k*-th and the $(k+1)$-th microfailure is denoted as $\Delta \varepsilon _{k+1}$. Every microfailure or extension of a microcrack in bone causes additional material degradation, so it is assumed that the expected values of strain between microfailures will conform to the relationship:

1 $$\begin{aligned} {\mathbb {E}}(\Delta \varepsilon _k) \ge {\mathbb {E}}(\Delta \varepsilon _{k+1}) \end{aligned}$$

This condition, as discussed in the “Appendix” (Sect. 5), implies that the process in question is a New-Better-than-Used-in-Expectation (NBUE) process. Indeed, the accumulation of a significant number of microfailures is anticipated to result in the degradation of structural integrity, i.e.,, the condition $\lim _{k\rightarrow \infty } \Delta \varepsilon _k = 0$ is expected to hold, as it is argued below. The latter relationship is interpreted as the observation that near the macroscopic failure, there is a huge accumulation of microfailures in a catastrophic process, which mathematically is represented by an explosive stochastic process (in a finite time, an enormous number of microfailures occur).

As in an ordinary Poisson process, in the proposed model the time between events follows an exponential probability distribution:

2 $$\begin{aligned} \text {Prob}(0 < \Delta \varepsilon _k \le u) = \lambda _k \text {exp}(\lambda _k u), \qquad {\mathbb {E}}(\Delta \varepsilon _k) = \frac{1}{\lambda _k} \end{aligned}$$

That is, $\Delta \varepsilon _k \sim \text {Exp}(\lambda _k)$. The rationale for this choice is that the exponential distribution is the only one with a constant *hazard rate function* (that is, the distribution is “memory-less”). After the occurrence of the *k*th microfailure, it is clear that the parameter $\lambda _k$ of the exponential distribution will inevitably decrease because of the embrittlement of the material following. There are many ways to choose $\lambda _k$, and each choice would define a different stochastic model. A straightforward option is utilized in the proposed model:

3 $$\begin{aligned} \lambda _k = \lambda \alpha ^{-k+1}, \quad \alpha < 1 \end{aligned}$$

This choice guarantees that biparametric stochastic process $\{N_{\varepsilon }\}_{\varepsilon \ge 0}$ is explosive and ensures that an arbitrarily large number of microfailures will occur, leading to mechanical failure for a finite value of the strain (which is what is observed in bone and all real materials). In addition, the choice is justified because it is well in agreement with the experimental data. For any other stochastic process of this type that is “memory-less” between the occurrence of microfractures, the so-called *deterioration rate* $\beta _k$ can be defined as:

4 $$\begin{aligned} \beta _k = - \ln \frac{\lambda _{k+1}}{\lambda _k} > 0 \end{aligned}$$

So this model is, in fact, a model with constant deterioration. Summarizing the above, the stochastic failure process is described by two parameters $(\alpha ,\lambda )$ and, formally, we have a stochastic process of the type:

5 $$\begin{aligned} \{(N_{\varepsilon },{\mathcal {F}}_{\varepsilon })|\varepsilon \in {\mathbb {R}}^{+}, N_{\varepsilon } \in {\mathbb {N}} \} \end{aligned}$$

where ${\mathcal {F}}_{\varepsilon }$ is the filtration of the $\sigma$-algebra of the stochastic process (see Sect. 5). For each value of $\varepsilon$, the set $(N_{\varepsilon },{\mathcal {F}}_{\varepsilon })$ is a random variable in ${\mathbb {R}}$. Mathematically, the model is a generalization of a stationary Poisson process with two interesting features: firstly, it is a doubly stochastic Poisson process (a Cox process) (Cox 1955; Grandell 1976; Chetlur and Dhillon 2018) and, secondly, it is an explosive stochastic process (an unbounded number of microfailures appear in a finite time) (Savits 1969; Greenberg 1991; Klüppelberg and Mikosch 1995; Anderson et al. 2018) (some additional technical aspects of the model are explained in detail only in the “Appendix” in Sect. 5).

#### Estimation of the parameters

The parameters ($\alpha ,\lambda$) of the proposed stochastic model are estimated by comparing the difference between the increments of the experimental strain with the increments of the expected strain. Specifically, $\alpha$ and $\lambda$ are obtained by minimizing a penalty function $\Phi (\alpha ,\lambda )$, which reaches its minimum for optimal parameter fitting:

6 $$\begin{aligned} \Phi (\alpha ,\lambda )= & {} \left[ \mu _{\infty }(\alpha ,\lambda ) - \sum _{i=1}^n \Delta \varepsilon ^*_i\right] ^2 \nonumber \\{} & {} + \sum _{k=1}^n \left[ \mu _{k,k+1}(\alpha ,\lambda ) - \Delta \varepsilon ^*_{k,k+1}\right] ^2 \end{aligned}$$

where $\mu _{\infty }(\alpha ,\lambda ) = {\mathbb {E}}\left( \sum _{k=1}^{\infty } \Delta \varepsilon _k\right)$ is the total expected strain from the first microfailure ($i=1$) to the last one ($i= n$), i.e., from the initial microfailure to the maximum strain, being *n* the number of strain increments up to complete failure, and the $\Delta \varepsilon ^*_i$ are the empiral increments between detected AE events. In addition, $\mu _{i,j} = {\mathbb {E}}\left( \sum _{k=i+1}^j \Delta \varepsilon _k\right)$ is the expected value of the strain increment between the microfailures *i* and *j*, and $\Delta \varepsilon _{i,j}^* = \varepsilon _j^* - \varepsilon _i^*$ is the corresponding experimental increase. The deduction from the above expected values is given in Sect. 5.1 of the “Appendix”, where we obtain:

7 $$\begin{aligned} \left\{ \begin{array}{rl} \mu _{\infty }(\alpha ,\lambda ) = {\mathbb {E}}\left( \sum _{i=1}^{\infty }\Delta \varepsilon _i\right) &{} = \frac{1}{(1-\alpha )\lambda }\\ {} &{}\\ \mu _{i,j}(\alpha ,\lambda ) = {\mathbb {E}}\left( \Delta \varepsilon _{i,j}\right) &{} = \frac{\alpha ^i-\alpha ^{j+1}}{(1-\alpha )\lambda } \end{array}\right. \end{aligned}$$

### Experimental setting

**Fig. 1:**
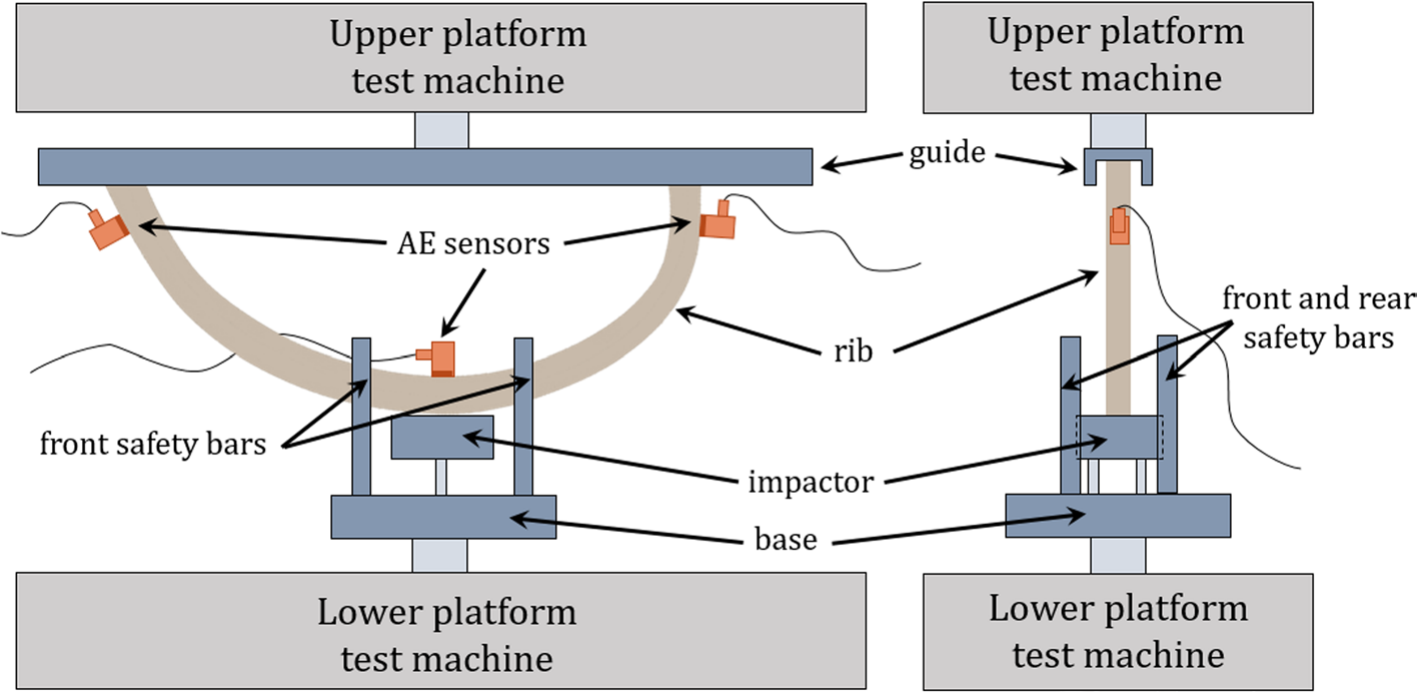
Experimental setting: the rib is placed with its extremes inside the guide and the outer central region in contact with the impactor. An AE sensor is placed in the central region where the main fractures occur along with two sensors placed near the ends to discriminate friction events

The model proposed in Sect. 2.2 was applied to a sample consisting of fifteen human complete ribs, which were subjected to a three-point bending test monitored by three AE sensors. Bending tests were performed with a ZwickRoell^®^ Proline 7.1 and a load cell HBM^®^, following the procedure of previous work (García-Vilana et al. 2022a). A U-shaped guide was placed on the upper platform into which the rib extremes were inserted (the rib was contained in the machine plane, see Fig. 1). The guide was previously covered with lubricant and the rib ends were wrapped with polytetrafluoroethylene band to ensure a low-friction sliding of the rib ends. On the lower part of the platform, a base was fixed in which the impactor was attached. The impactor exerted the force on the central external region of the rib. Due to end sliding, a few AE events occur, associated with friction, which are detected by one of the ends. These events are removed from the sample of AE events, as they can be perfectly distinguished from AE events associated with microfailures based on the amount of energy involved.

All the tests were video-recorded with a high-speed camera (PCO^®^ 1200s) and from the sequence of video frames, the displacements on the rib during the test were determined following a Digital Image Correlation (DIC) procedure with Matlab^®^. To find the displacement vector field, a DIC-motion-tracking algorithm was used to calculate the displacements of different material points along the rib and located in the plane in which the rib was bending (all the details were described in a previous work (Velázquez-Ameijide et al. 2021)). From the displacement field the change of rib curvature was computed, as explained in another previous work (García-Vilana et al. 2022a, 2022b), which, in turn, allowed us to compute the strain tensor.

### Acoustic emission measures

AE is a nondestructive control technique that does not involve any energy input into the materials (Ciaburro and Iannace 2022). When a material medium is internally stressed, the microstructure is deformed, and the mechanical work done by external forces to deform the medium is accumulated as elastic potential energy. Due to the lack of complete homogeneity in bone microstructure, some stress concentrations can be discerned at the microscopic level. When the stress concentrations exceed a specific threshold, they produce localized microdamage in the microstructure (Conward and Samuel 2023). Then, the internal stress of the bone tissue is alleviated locally as a result of microcracking, and some energy stored locally as elastic potential energy is radiated as elastic waves (Lysak 1994). These waves are typically ultrasonic signals emitted spontaneously from the stress concentration points. Therefore, through the utilization of multiple AE sensors to triangulate the signals, it is possible to approximate the localization and magnitude of the microdefects based on the total energy detected (Ciaburro and Iannace 2022; Ono 2018).

Although attenuation determination is important in large engineering components (Ono 2018), in the present case, ribs of a few centimeters were used, so the attenuation is generally small, and each signal is detected by two or more sensors located along the sample (in our experimental test, we used two guard sensors at the ends and a central one close to the maximum stress region).

All the tests described in Sect. 2.3 are conducted monitoring the occurrence of AE events by means of three resonant AE sensors (VS700-D, Vallen System Gmbh) which were placed along the rib: two sensors at the ends to discriminate friction signals and another sensor in the inner central region of the rib (see Fig. 1), where greater stresses and macroscopic fracture occur. Three AE amplifiers (AEP4) were used together with a four channel system (AMSY-5) and a band-pass filter between 25 and 1100 kHz to discriminate noise or possible friction signals. In addition, a lighting system was installed in the camera and its voltage was connected to both force and AE acquisition systems. Prior to the force increase, the light was interrupted for a very short time, and the acquisition systems recorded null voltages during this time. Thus, based on the recorded voltage drop and the video light switch-off, forces, displacements and AE signals were synchronized, with a precision better than 10 ms.

### Statistical analysis

The data obtain from the experimental tests and the stochastic model fitting were analyzed using statistical tools. Specifically, the influence of some variables or parameters (as age) on the results obtained were analyzed using linear regression analysis. A *p* value$<0.05$ was considered as statistically significant.

Moreover, the XLSTAT^®^ statistical package was used for the distribution fittings of some data obtained.

Finally, the Principal Component Analysis (PCA) was used to explore the correlations between parameters and variables, as well as to describe the variability of the data set.

## Results and discussion

In this section, the results obtained from the fifteen human rib bending tests, monitored with acoustic emission technique, and the of the stochastic failure model fitting to the experimental data are presented and discussed.

### Quantity and energy of AE events

**Fig. 2:**
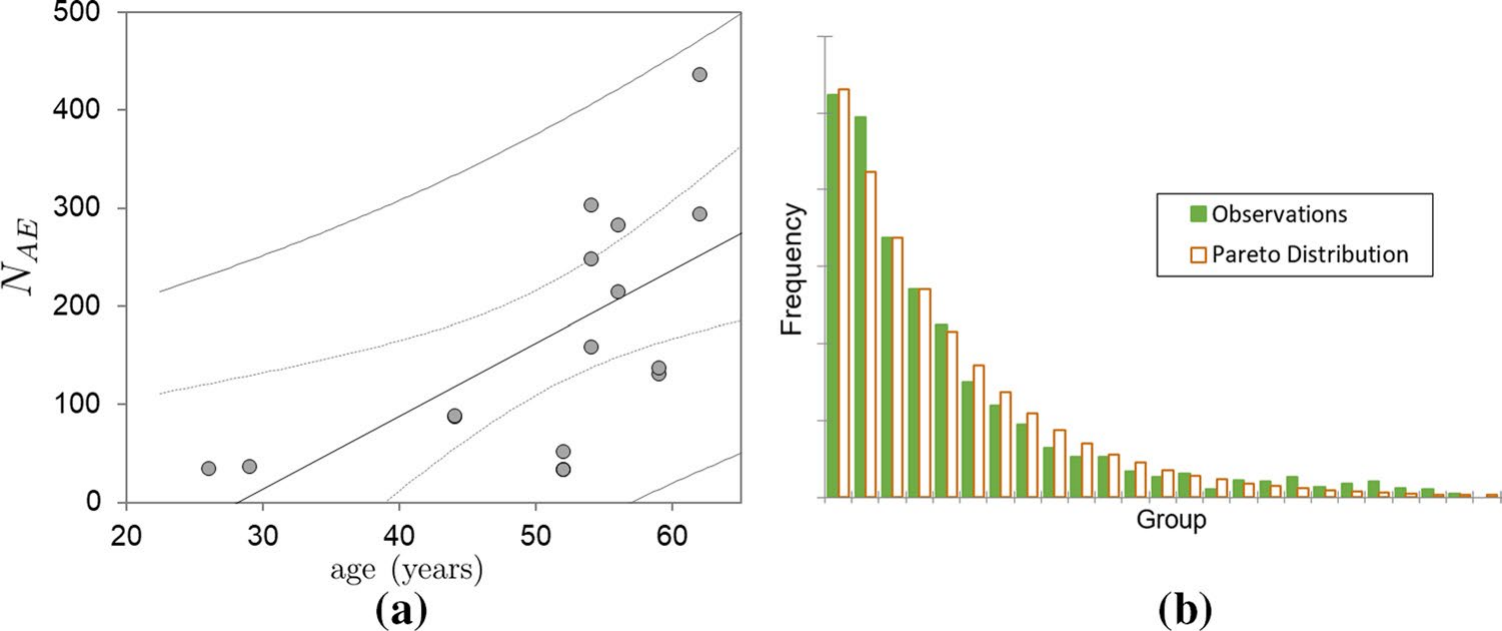
**a** Number of detected AE events $N_{AE}$ versus age ($p<0.0075$), **b** Probability Distribution Function of relative energy for a specific specimen

**Fig. 3:**
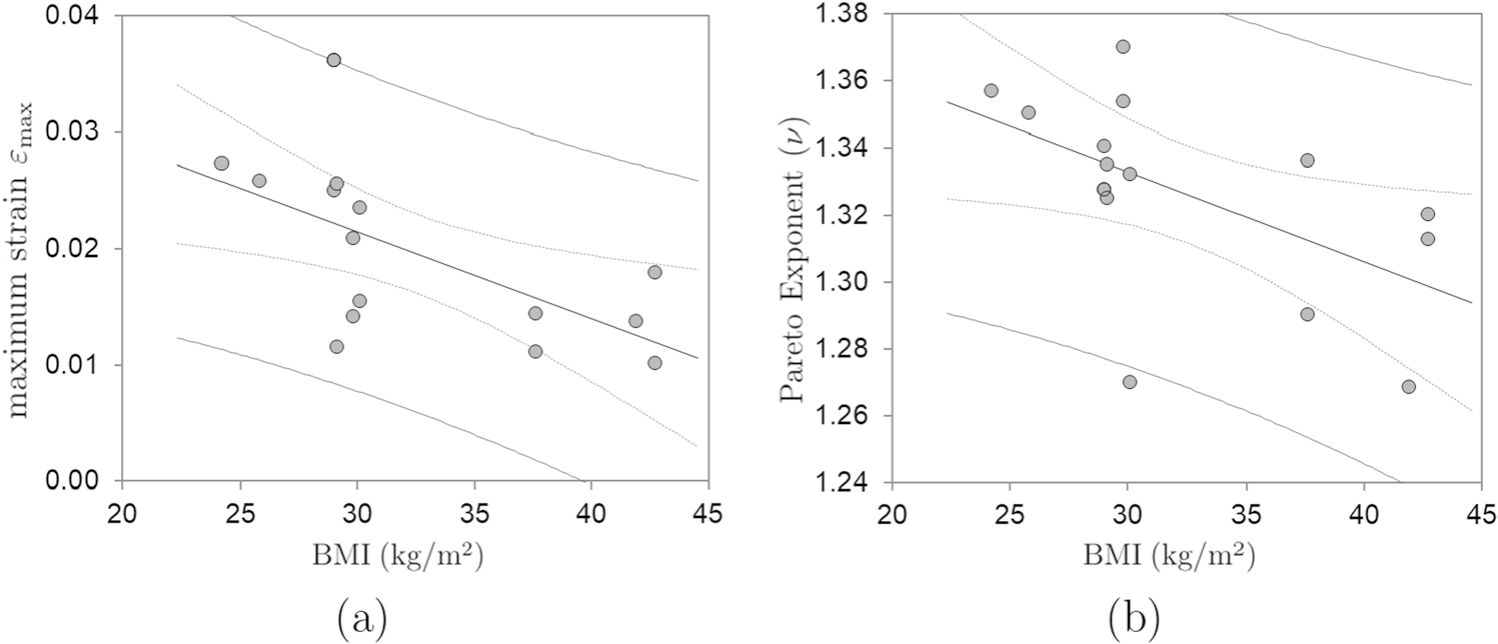
**a** Measured maximum strain $\varepsilon _{\max }$ versus BMI ($p<0.015$), b Computed Pareto exponent for AE energies versus BMI ($p< 0.035$)

The detected number of AE events ($N_{AE}$) for each human rib specimen is quite variable. An interesting empirical finding is that there is a significant positive correlation (*p* value <0.0075) between $N_{AE}$ and the age of the PMHS; specifically, a greater number of events are detected in elderly PMHSs. Furthermore, AE signal analysis reveals a Pareto distribution in the energies of the set of EA events for each rib specimen:

8 $$\begin{aligned} \text {Prob}(\text {energy an AE signal} \le E) = 1 - \left( \frac{E_m}{E} \right) ^\nu \end{aligned}$$

where $E_m$ denotes the minimum energy, which is chosen to exclude residual and friction-related signals, and $\nu > 1$ is the Pareto exponent. Prior research has documented this distribution pattern in relation to additional biological tissues (Sánchez-Molina et al. 2015; Baró et al. 2016; García Vilana et al. 2016a, b). The number of AE events as a function of age and the typical relative energy distribution for a representative specimen are both illustrated in Fig. 2. Furthermore, it has been observed that the PMHS BMI significantly influences the maximum strain $\varepsilon _{\max }$ (*p* value $< 0.015$), and the average energy of the signals, as well as the Pareto exponent $\nu$ (*p* value $< 0.035$). The scatter plot of these two magnitudes versus BMI is illustrated in Fig. 3.

### Fitting to the stochastic model of microfailures

**Fig. 4:**
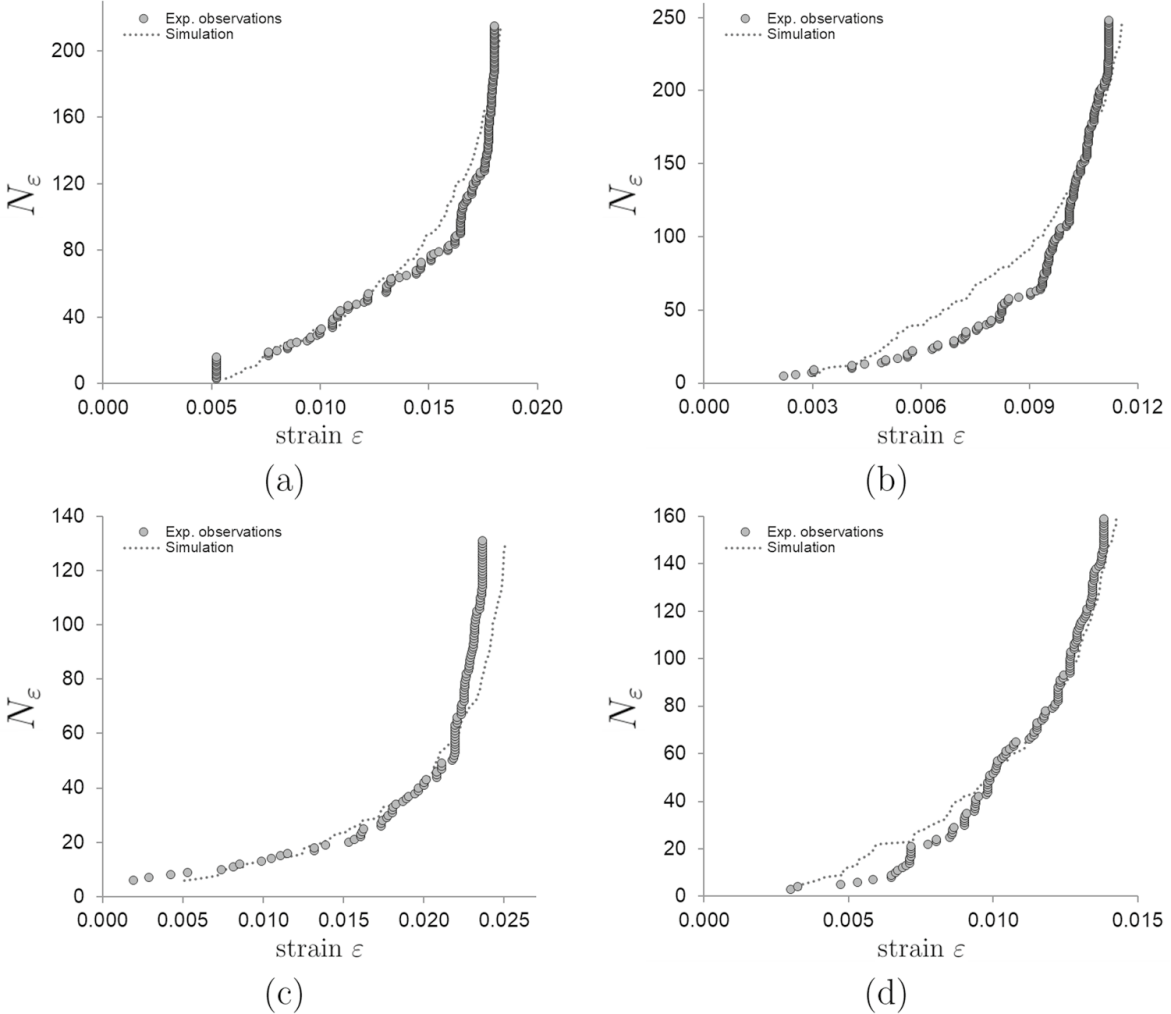
Comparison of the AE events observed and simulated as a function of strain $\varepsilon$, for four specimens: **a** specimen 06R, **b** specimen 09 L, **c** specimen 13 L, **d** specimen 15 L. The curves for the remaining eleven specimens are qualitatively similar. Experimental observations are denoted by gray circles in each of the four graphs. The observations are utilized to estimate the parameter values of the model, which are then implemented in the simulation of analogous curves denoted by a dashed line. While the simulation and observations exhibit greater concordance in the vertical asymptote, their divergence becomes more pronounced during the initial stages as a result of the reduced quantity of stochastic accumulated events. The simulated curves, despite sharing the identical parameters of the original curve, have a minimal likelihood of being identical to the original due to the stochastic nature of the model. On the contrary, a notable similarity between the simulated and original curves is observed, particularly in the vicinity of the asymptote

This section shows how the distribution of detected microfailures resembles a simulated instance of the stochastic process described in Sect. 2.2. The parameters $(\alpha , \lambda )$ of each specimen were determined by minimizing the function defined in (6). Figure 4 depicts the observations used for fitting as well as an additional simulation with the same parameters (since each simulation is a stochastic simulation, even for a fixed value of the parameters, generates a slightly different curve, so the original curve is not expected to be reproduced).

The $\alpha$ parameter is significantly correlated with age (*p* value = 0.034) and BMI (*p* value = 0.028), whereas the $\lambda$ parameter is very significantly correlated with BMI (*p* value = 0.005) (it has been verified that there is no significant correlation between age and BMI in the sample used). The parameters $\alpha$ and $\lambda$, on the other hand, are not independent of each other, but have a positive and significant correlation (*r* = 0.822, *p* value $<0.0001$). In addition, defining the (random) variable $\varepsilon _{\infty }:= \lim _{n\rightarrow \infty } \sum _{k=1}^n \Delta \varepsilon _k$, we have that the correlation between its expected value ${\mathbb {E}}(\varepsilon _{\infty })$ and the observed value for the maximum strain ($\varepsilon _{\max }$) is very high (*r* = 0.975, *p* value <0.0001).

### Analysis of calculated parameters

For determining the number of underlying factors (components) required to account for the observed variability and the internal correlations between the calculated parameters ($\alpha , \lambda , \nu$) and the measured variables ($N_{AE}, \varepsilon _{\max }$, age, BMI), a Principal Component Analysis (PCA) was performed. Table 1 provides an overview of the found relationships by displaying the correlation matrix between the calculated and measured variables, which reveals significant associations between all the magnitudes.

**Table 1:** Spearman correlation coefficients and *p* values

	${\alpha }$	${\lambda }$	${\nu }$	${N_{AE}}$	${\varepsilon _{\max }}$	Age	BMI
$${\alpha }$$	–	$$0.822^{***}$$	$$-0.421$$	$$-0.782^{***}$$	$$-0.557^{*}$$	$$0.556^*$$	$$0.575^*$$
$${\lambda }$$	$$(<0.0001)$$	–	$$-$$0.425	$$0.882^{***}$$	$$-0.807^{***}$$	0.488	$$0.698^{**}$$
${\nu }$	(0.119)	(0.116)	–	$-$0.454	0.446	$-0.634^*$	$-0.641^*$
$${N_{AE}}$$	(0.001)	$$(<0.0001)$$	(0.092)	–	$$-0.707^{***}$$	$$0.758^{***}$$	$$0.603^*$$
$${\varepsilon _{\max }}$$	(0.034)	(0.0004)	(0.097)	(0.004)	–	$$-0.387$$	$$-0.738^{***}$$
Age	(0.034)	(0.067)	(0.013)	(0.002)	(0.155)	–	0.454
BMI	(0.028)	(0.005)	(0.012)	(0.020)	(0.002)	(0.091)	—

*Significance levels:* $${}^*< 0.05,^{**}< 0.01,^{***} <0.005$$

*On the diagonal:* Spearman’s correlation coefficients

*Under the diagonal:* *p* values in parentheses

The data in the sample, presented in Table 1, show that there is a significant negative correlation of $\lambda$ and $\alpha$ with the ultimate failure strain $\varepsilon _{\max }$. This means that increased microfailure occurrence rate $(1/\lambda )$ and deterioration rate $(\beta = -\ln \alpha )$ result in a reduced ultimate failure strain, i.e.,, the specimen exhibiting high values of $(\alpha ,\lambda )$ is incapable of undergoing significant strain prior to fracture. Additionally, Table 1 shows that overweight (BMI) is associated with an increase in both $\alpha$ and $\lambda$, which may account for the fact that individuals with a higher BMI have a lower failure strain (*p* value $\approx$0.002), as was pointed out in recent studies (Velázquez-Ameijide et al. 2020, 2021). The correlation between age and $\alpha$ is statistically significant; thus, the deterioration rate increases with age. Finally, the Pareto exponent ($\nu$), which governs the energy of EA events, exhibits a marginally significant negative correlation with both age and BMI. However, an intuitive explanation for this result has yet to be identified.

**Fig. 5:**
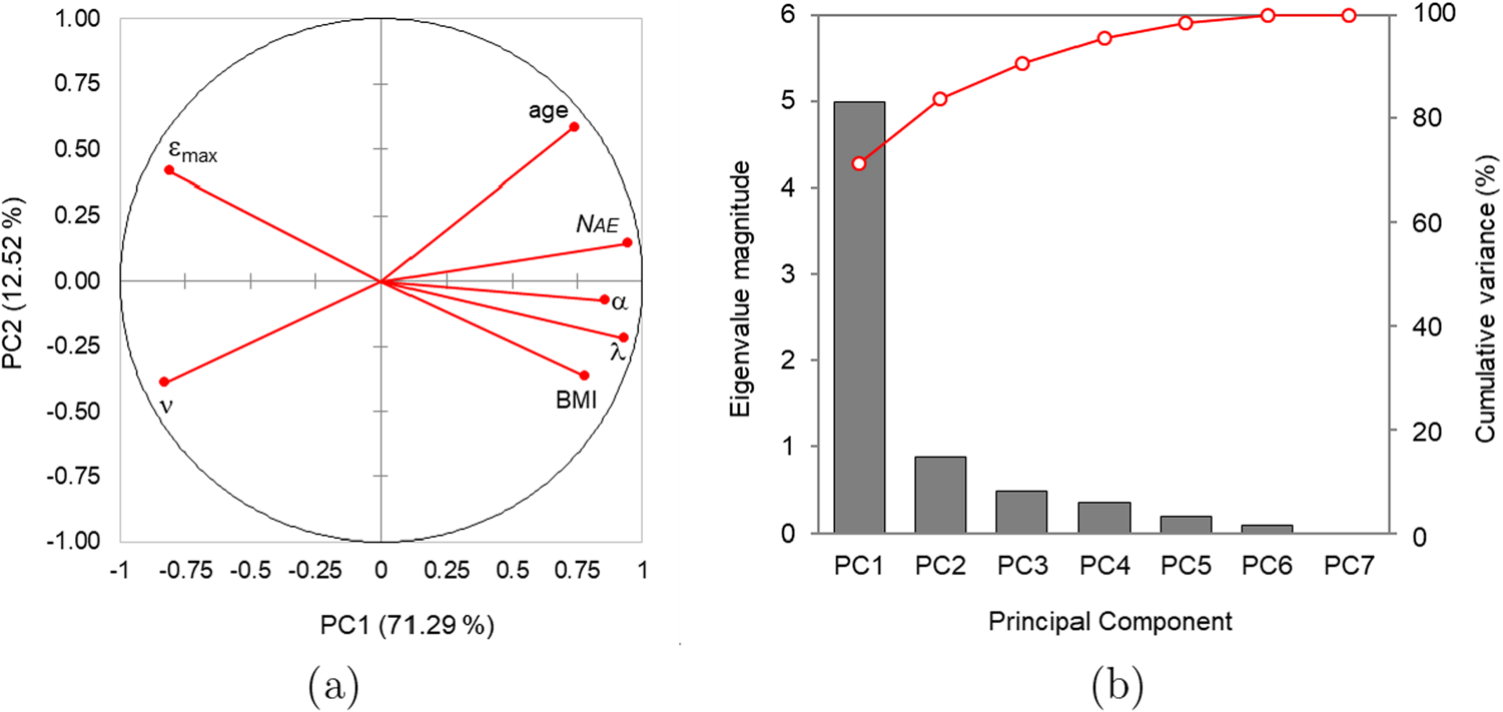
**a** Correlation diagram of the computed parameters and parameters with PC_1_ (*inveteration* factor) and PC_2_: the horizontal axis represents the correlation with PC_1_ and the vertical axis with PC_2_. As can be seen, most magnitudes are highly correlated with PC_1_, while PC_2_ exhibits marginal correlations with the magnitudes studied. **b** Absolute and relative weights of the principal components in the observed variability, it can be seen that PC_1_ has a much greater weight than the rest of the possible factors together

Since the calculated parameters ($\alpha , \lambda , \nu$) and the observed variables ($N_{AE}, \varepsilon _{\max }$, age, BMI) are not statistically independent, it is worth considering what factors explain their covariability. The specific goal of PCA in this case is to determine the number of underlying factors needed to account for the covariability observed in the seven original magnitudes. A PCA of seven magnitudes, by construction, represents them as combinations of seven statistically independent random variables PC_1_ to PC_7_. By diagonalization of the covariance matrix, these new random variables are obtained as linear combinations of the original ones. The PCA performed on our dataset reveals that the first component PC_1_ accounts for 71.3% of the observed variability (see Fig. 5b). This indicates that a single underlying factor can accurately forecast the values of the seven magnitudes under consideration for each specimen. The second component PC_2_ is much less important, accounting for a mere 12.5% of the observed variability and the remaining five factors contributed marginally to the observed variability and are regarded as residuals. Hence, it seems that the first component (PC_1_) is linked to a biological condition that influences the observed or measured mechanical properties and the computed parameters. As shown in Fig. 5a, this component PC_1_ is significantly correlated with all the quantities examined. Because in all specimens PC_1_ increases with age, affects the loss of toughness and decreases the maximum strain, it seems to be a process associated with aging and the degradation of mechanical properties, so we will call it *inveteration* (from Latin *inveterare* ‘to mature, to make look old, to inure’). The inveteration factor can be estimated quite accurately ($r^2 = 0.765$) by means of the subsequent equation:

9 $$\begin{aligned} \text {PC}_1 = -9.748 + 0.079 \cdot \text {age} + 0.176 \cdot \text {BMI} \end{aligned}$$

Both anthropometric variables exert an influence: age is statistically significant (*p* value = 0.027), and body mass index (BMI) is even more so (*p* value = 0.007). Thus, a practitioner could estimate the PC$_1$ value -or inveteration- using just the patient’s age and BMI. In contrast, the correlation between PC$_2$ and the remaining parameters and variables is marginal, making it difficult to identify an underlying factor that explains the value of this component.

## Conclusion

This study shows that AE-measured microfailures appear randomly and without a fixed or discernible pattern, but can be modeled adequately with a stochastic failure model. The proposed model can be mathematically described as a Cox process that is explosive (Coxplosive process), which is also a New-Better-than-Used-in-Expectation (NBUE) failure process. The model delineates the manner in which the degradation of bone material by each microfailure increases the probability of subsequent microfailures occurring. Eventually, this degradation culminates in a catastrophic avalanche of chain microfailures, which leads to the macroscopic fracture of the bone.

All the variables measured, as well as the parameters of the stochastic model, exhibit significant correlations with anthropometric variables such as age or body mass index (BMI). In particular, age seems to play an important role in the number of AE events $(N_{AE})$ and the parameter $\alpha$ that gives the acceleration of bone deterioration. This implies that older individuals are more susceptible to microfailures and experience a more noticeable deterioration rate, as indicated by a lower failure strain ($\varepsilon _{\max }$) and reduced toughness (Velázquez-Ameijide et al. 2021; Katzenberger et al. 2020; Larsson et al. 2021). On the other hand, the two parameters computed to represent the distribution of AE event occurrences, namely $(\lambda , \nu )$, which exhibit no significant correlation with age, do exhibit a significant correlation with BMI. This shows that all the calculated parameters $(\alpha , \lambda , \nu )$ and the two observed variables $(N_{AE},\varepsilon _{\max })$ in every case exhibit significant correlations with the anthropometric variables, age or BMI, and in some cases with both. This indicates that biological mechanisms associated with age and overweight exert substantial control over each of these variables in a significant way. This is especially important for calculated variables that represent abstract parameters but capture the effect of biological mechanisms; otherwise, values would be obtained that are unrelated to anthropometric variables.

## Data Availability

A dataset with all the computations and analyses conducted will be available publicly in the web CORA-RDR [https://dataverse.csuc.cat/].

## Appendix

The proposed stochastic model is defined on a *probability space* $\Omega _{{\mathbb {P}}} = (\Omega ,{\mathcal {A}},{\mathbb {P}})$, where $\Omega$ is the set of all elementary events or *sample space*; ${\mathcal {A}}\subset {\mathcal {P}}(\Omega )$ is the *event space*, a collection of subsets of $\Omega$ (by logical requirements associated with Boolean operations, ${\mathcal {A}}$ has a structure of $\sigma -$algebra of sets); and, finally, ${\mathbb {P}}$ is a *probability measure* such that $0 \le {\mathbb {P}}(A) \le 1$ for $A\in {\mathcal {A}}$, having as particular values ${\mathbb {P}}(\varnothing ) = 0$ and ${\mathbb {P}}(\Omega ) = 1$. A *simple random variable* is a measurable function $X:\Omega \rightarrow {\mathbb {R}}$. The probability that the values of a random variable fall within the range $[x_1,x_2]$ is given by:

10 $$\begin{aligned} \text {Prob}(x_1 \le X \le x_2) = {\mathbb {P}}(\{\omega \in \Omega | X(\omega ) \in [x_1,x_2] \}) \end{aligned}$$

The set of all simple random variables is denoted as ${\mathbb {R}}^\Omega$. Under these conditions, a *simple stochastic process* is characterized by a collection of random variables $\{X_u\in {\mathbb {R}}^\Omega | u\in [0,U]\subset {\mathbb {R}}^+\}$. Random variables and stochastic processes can be made *n*-dimensional through the combination of simple stochastic processes and random variables, since ${\mathbb {R}}^\Omega \times {\mathbb {R}}^\Omega \simeq ({\mathbb {R}}^2)^\Omega$, and so forth.

Given a stochastic process, an associated *filtration* can be defined as an indexed family $\{ {\mathcal {F}}_\gamma \}_{\gamma \ge 0}$ with ${\mathcal {F}}_\gamma \subseteq {\mathcal {F}}$ of $\sigma$-algebras, where the index $\gamma \in {\mathbb {R}}^+$ is subject to the condition that if $\gamma _1 \le \gamma _2$, then ${\mathcal {F}}_{\gamma _1} \subseteq {\mathcal {F}}_{\gamma _2}$. Subsequently, a *filtered probability space* $\left( \Omega , {\mathcal {F}}, \left\{ {\mathcal {F}}_{t}\right\} _{t\ge 0}, {\mathbb {P}}\right)$ is a probability space equipped with the filtration $\{ {\mathcal {F}}_\gamma \}_{\gamma \ge 0}$. Filtered probability spaces are typically characterized as *complete* (i.e.,, ${\mathcal {F}}_0$ contains all ${\mathbb {P}}$-null sets (zero-measure sets), and *right-continuous* (i.e., ${\mathcal {F}}_\gamma = {\mathcal {F}}_{\gamma +}:= \bigcap _{\delta > \gamma } {\mathcal {F}}_\delta$ for all $\gamma$).

In *realibility theory*, non-homogeneous renewal process are used to model the accumulated damage to a system that is subjected to both shocks and repairs. Specifically, the process $N = \{N_{\varepsilon }| \varepsilon > 0, N_{\varepsilon }(\omega )\in {\mathbb {N}} \}$. Let $\varepsilon _n$ be its first-passage time to level *n*, i.e., $\varepsilon _n = \inf \{\varepsilon> 0| N_{\varepsilon }(\omega ) > n\}$, then $\varepsilon _n$ is said to have New-Better-than-Used-in-Expectation (NBUE) distribution, if:

11 $$\begin{aligned} {\mathbb {E}}(\varepsilon _n-\varepsilon ^*|\varepsilon _n>\varepsilon ^*) \le {\mathbb {E}}(\varepsilon _n) \end{aligned}$$

where ${\mathbb {E}}(R|f\in A)$ is the conditioned expectation for the random variable *R*, given the event that $f(\omega )\in A\subset {\mathbb {R}}$ (Lam 1992; Belzunce et al. 2002). It can be shown that the stochastic process $\{N_{\varepsilon }\}_{\varepsilon \ge 0}$, in fact, is an NBUE process, by the condition (1).

### Expected values

In this section, the expected values for the model fitting in equation (6) are computed. Let $\varepsilon _{\max }$ be the strain increase from the first microfailure detected to the last one, i.e.,, $\varepsilon _{\max } = \sum _{k=1}^{n_{\max }} \Delta \varepsilon _k \approx \sum _{i=1}^\infty \Delta \varepsilon _i = \varepsilon _{\infty }$; then, a straightforward computation, using (2) and (3), allows us to calculate the expected value:

12 $$\begin{aligned} {\mathbb {E}}(\varepsilon _{\infty })= & {} {\mathbb {E}}\left( \sum _{k=1}^{\infty }\Delta {\varepsilon }_k\right) = \sum _{k=1}^{\infty }{\mathbb {E}}(\Delta {\varepsilon }_k) = \sum _{k=1}^{\infty }\frac{1}{\lambda _k}\nonumber \\= & {} \sum _{k=1}^{\infty }\frac{\alpha ^{k-1}}{\lambda }=\frac{1}{(1-\alpha )\lambda } \end{aligned}$$

Likewise, the increase in strain from the *i*-th event and the *j*-th event, denoted by $\Delta \varepsilon _{i,j}$, is:

13 $$\begin{aligned} {\mathbb {E}}(\varepsilon _{i,j})= & {} {\mathbb {E}}\left( \sum _{k=i}^{j}\Delta {\varepsilon }_k\right) = \sum _{k=i}^{j}{\mathbb {E}}(\Delta {\varepsilon }_k) \nonumber \\ {}= & {} \sum _{k=i}^{j}\frac{\alpha ^{k-1}}{\lambda }=\frac{\alpha ^{i-1}-\alpha ^j}{(1-\alpha )\lambda } \end{aligned}$$

In a similar vein, variances can be computed. Take the calculation of ${\mathbb {E}}(\varepsilon _{\infty }^2)$:

$$\begin{aligned} {\mathbb {E}}(\varepsilon _{\infty }^2)= & {} {\mathbb {E}}\left( \left[ \sum _{k=1}^{\infty }\Delta {\varepsilon }_k\right] ^2\right) \\= & {} {\mathbb {E}}\left( \sum _{k=1}^{\infty }\sum _{l=1}^k \Delta {\varepsilon }_{k-l+1} \Delta {\varepsilon }_l \right) \\= & {} \sum _{k=1}^{\infty }\sum _{l=1}^k {\mathbb {E}}(\Delta {\varepsilon }_{k-l+1} \Delta {\varepsilon }_l) \\= & {} \sum _{k=1}^{\infty } \left[ {{\hat{\sum }}_{l=1}^k} {\mathbb {E}}(\Delta {\varepsilon }_{k-l+1} \Delta {\varepsilon }_l) + {\mathbb {E}}(\Delta {\varepsilon }_{k}^2)\right] \\= & {} \sum _{k=1}^\infty \left( k - \text {od}_k\right) \frac{\alpha ^{k-1}}{\lambda ^2} + \sum _{k=1}^\infty \frac{2\alpha ^{2k-1}}{\lambda ^2}\\= & {} \frac{2\alpha ^2\lambda ^{-2}}{(1-\alpha )(1-\alpha ^2)} + \frac{2\alpha \lambda ^{-2}}{1-\alpha ^2} = \frac{2\alpha \lambda ^{-2}}{(1-\alpha )(1-\alpha ^2)} \end{aligned}$$

where the special summation $${\hat{\sum }}_{l=1}^k$$ omits the terms where $$k-l+1=l$$, which are considered separately with in the second summation. The value of the term od$$_k$$ is determined by whether *k* is odd (od$$_k = 1$$) or even (od$$_k = 0)$$ [for example, od$$_k = \sin ^2(\pi k/2)$$].

Then, considering that $\text {Var}(\varepsilon _{\infty }) = {\mathbb {E}}(\varepsilon _{\infty }^2)-{\mathbb {E}}(\varepsilon _{\infty })^2$, we can compute this variance as:

14 $$\begin{aligned} \text {Var}(\varepsilon _{\infty }) = \frac{2\alpha \lambda ^{-2}}{(1-\alpha )(1-\alpha ^2)} - \frac{\lambda ^{-2}}{(1-\alpha )^2} = \frac{1}{(1-\alpha ^2)\lambda ^2} \end{aligned}$$

## Abbreviations

AE: Acoustic emission
BMI: Body Mass Index
DIC: Digital image correlation
PC(A): Principal component (analysis)
PMHS: Post mortem human subject
